# Effect of animal-sourced bioactive peptides on the in vitro development of mouse preantral follicles

**DOI:** 10.1186/s13048-020-00695-8

**Published:** 2020-09-15

**Authors:** Gang Liu, Shubin Li, Jinyu Ren, Chunyu Wang, Yaxuan Zhang, Xiulan Su, Yanfeng Dai

**Affiliations:** 1 Key Laboratory of Medical Cell Biology, Clinical Medicine Research Center, Affiliated Hospital of Inner Mongolia Medical College, 1 Tongdao North Street, Hohhot, 010050 Inner Mongolia China; 2 College of Life Science, Inner Mongolia University, 235 West Univ. Road, Hohhot, 010021 Inner Mongolia China

**Keywords:** Preantral follicles, BAPT, ROS, Embryonic development

## Abstract

The aim of this study was to investigate the effect of bioactive peptides (BAPT) from animal sources on the development of mouse preantral follicles in vitro. Preantral follicles were isolated and randomly divided into the following groups: an untreated group (control) and three groups supplemented with 20, 40 and 60 μg/mL BAPT, respectively. After establishing the in vitro follicle culture, the gene expression levels and hormone levels were quantified. After in vitro maturation, the developmental rates, reactive oxygen species (ROS) production levels and mitochondrial distributions of MII oocytes were investigated, followed by the analyses of embryonic developmental rates after in vitro fertilization.

The results showed that BAPT promoted the growth of mouse preantral follicles. Notably, after 14 d of in vitro culture, the levels of 17 β-estradiol and progesterone were up-regulated with BAPT treatments. Moreover, the expression levels of *Oct4*, *Bmp15*, *GDF9*, *FOXO3*, *Zp3*, *FOXL2*, *Inhibin alpha, SOD2*, *Catalase*, *GPx* and *Bcl-2* in the developing follicles were significantly up-regulated after BAPT treatments (*P* < 0.05), while BAPT significantly inhibited the expression levels of *BAX* (*P* < 0.05). Following BAPT treatments, the ROS production levels of MII oocytes were decreased while the mitochondrial distributions were significantly enhanced. Furthermore, increased maturation rates, fertilization and embryonic developmental rates were found in these BAPT-treated groups (*P* < 0.05).

These results demonstrated that BAPT significantly improved the development of preantral follicles in vitro by reducing ROS-dependent cellular damages and by enhancing mitochondrial distributions, thereby promoting the further applications of animal-derived BAPT in biomedical research.

## Introduction

Folliculogenesis, a major issue related with mammalian ovulation and fertility, occurs as a complex and dynamic process within female ovaries, which could be affected by multiple endocrine and intraovarian paracrine factors including antioxidants, nutrients and vitamins [1]. The dysfunction of mammalian folliculogenesis, due to abnormalities in endocrine/paracrine signals, metabolic dysfunction, impaired oocyte developmental abilities or altered gene expression patterns*,* results in infertility-related diseases and reproductive disorders, such as ovarian cancer and polycystic ovary syndrome (PCOS) [2, 3].

Due to the progressing development of assisted reproductive biotechnologies, these culture and maturation models of follicles mimic the native environmental differences of the developing follicles in vitro to obtain the functional germ cells [4]. Moreover, the culture models of follicles in vitro allow the investigation of specific regulatory mechanisms related with in vivo folliculogenesis, as well as the search of diverse supplementary factors to sustain follicular development and the assessment of therapeutic agents against various reproductive disorders [5, 6].

However, the production and accumulation of reactive oxygen species (ROS) from cellular metabolic processes in the developing follicles affects the development and survival of follicles during long-term culture [7]. Indeed, when excessive ROS levels overwhelm the balance of the cellular antioxidant defense system, oxidative stress (OS) occurs [8, 9], resulting in direct or indirect ROS-mediated damage to nucleic DNA, proteins and lipids, thereby altering their functions and resulting in the abnormal gene expression patterns, DNA double strand break (DSB) repair, chromosomal errors and follicular development [10–13].

Therefore, the addition of antioxidants to follicle cultures as ROS scavengers could prevent or decrease harmful damages, resulting in increased qualitiy of germ cells [14–16]. Although different antioxidants, such as rutin [17], β-mercaptoethanol [18] and α lipoic acid [19], have been routinely applied to the culture system of developing follicles, the effect of bioactive peptides (BAPT), natural antioxidants derived from various food or animals sources [20] on the development of mammalian preantral follicles has not yet been investigated.

BAPT were characterized as unique bioactive polypeptidic compounds with short sequences of approximately several or tens of amino acids [21, 22]. With the rapid development of purification techniques including membrane filtration [23], gel filtration chromatography (GFC) [24], Ion-exchange chromatography (IEX) [25], ultra-high-performance liquid chromatography-tandem mass spectrometry (UHPLC-MS/MS) [26] and rapid resolution liquid chromatography-tandem mass spectrometry (RRLC-MS) [27], abundant BAPT species were purified and identified, leading to the various applications of BAPT, ranging from the development of biomaterials to therapeutic uses, in the past decade [28].

Furthermore, numerous studies have revealed that the biological activities of BAPT, including antioxidants, hormonal effects, immune system regulation, anti-thrombotic, anti-hypertensive, anti-bacterial, anti-viral and anti-cancer effects [29], depended on the original species, amino acid composition, and sequence of BAPT [30]. For instance, our previous studies demonstrated that animal-derived BAPT could suppressed the progression of colorectal cancer [31] and gastric cancer [32] in vivo without damages to normal cells. Nevertheless, little is known about the possible antioxidant effects of BAPT on the mammalian reproductive system and follicle developmental proficiency.

Therefore, this study examined the effects of BAPT on the development of mouse preantral follicles and oocyte in vitro to assess the antioxidant effect of BPAT on the mammalian reproductive system.

## Results

The effect of BAPT on the development of preantral follicles cultured in vitro*.*

**Fig. 1:**
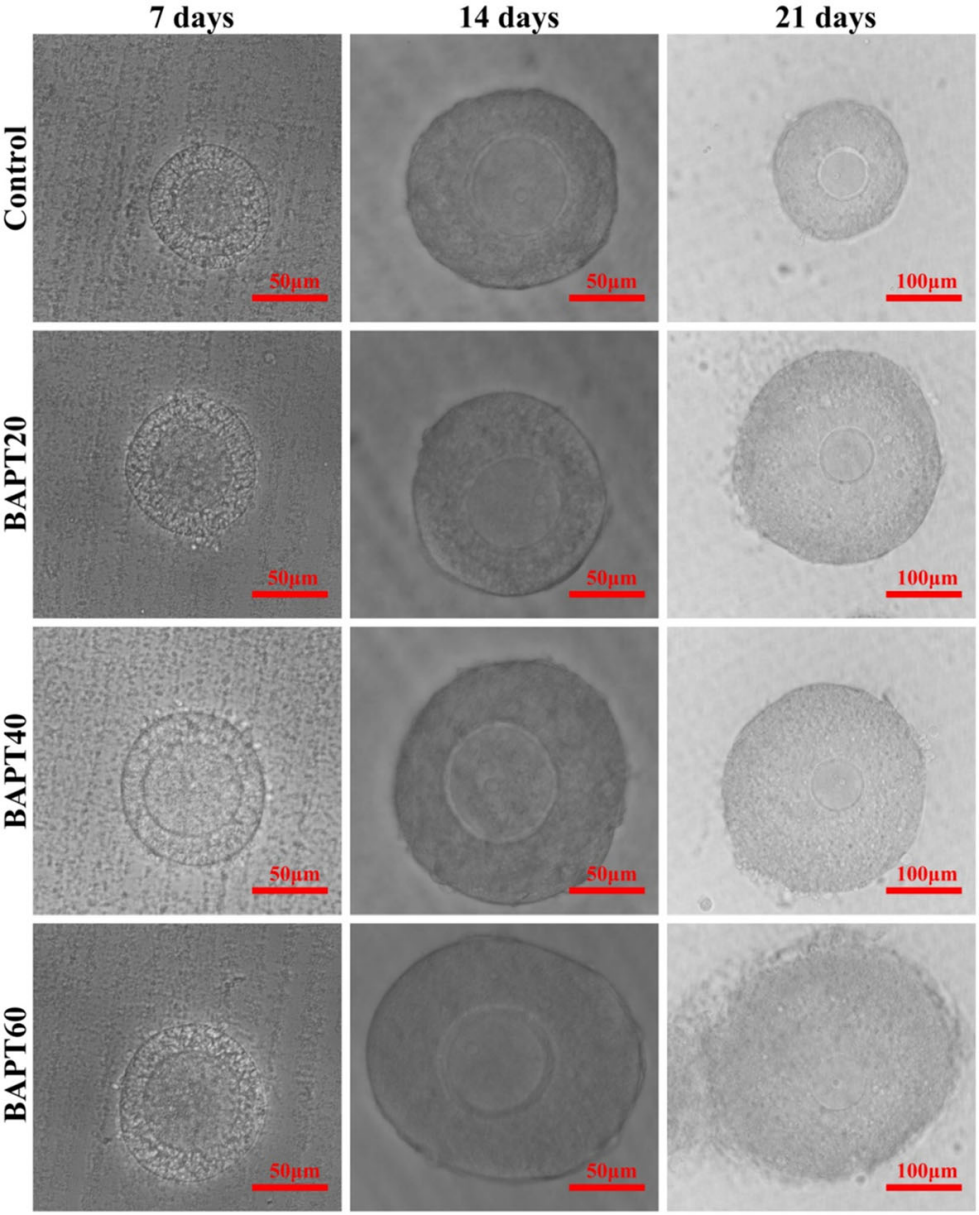
The representative growth of follicles cultured in vitro after different periods

**Fig. 2:**
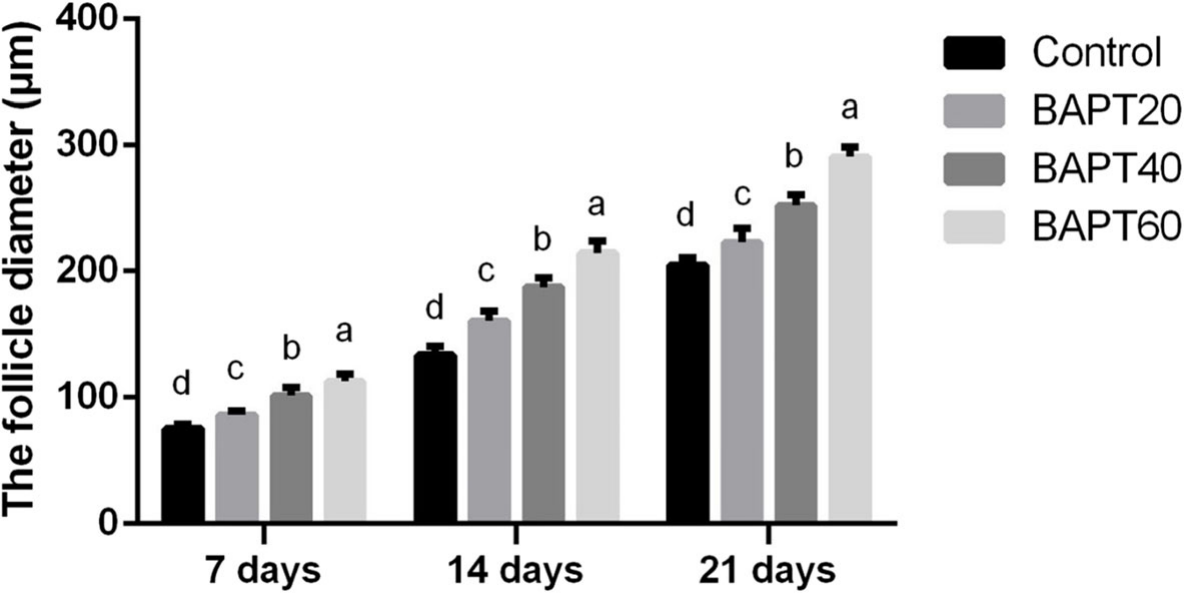
The mean diameter of follicles cultured in vitro after different periods. Note: In each panel, labeling with the different letter in each column indicates significant differences between different groups (*P* < 0.05)

To investigate the potential effect of BAPT treatment on the development of preantral follicles in vitro, the morphology and mean diameters of follicles cultured in vitro for 7, 14 and 21 d after BATP treatment were analyzed. As shown in Fig. 1 and Fig. 2, the results of follicular development in vitro showed that after cultured for 7 d, the growth of isolated follicles was significantly promoted in all groups with the mean diameters of cultured follicles significant increased from 75.11 ± 3.26 μm in the control group, 86.22 ± 3.38 μm in the BAPT 20 group, 101.56 ± 6.14 μm in the BAPT 40 group to 112.78 ± 6.20 μm in the BAPT 60 group (*P* < 0.05), respectively.

On day 14, the diameter of follicles significantly increased from 133.66 ± 6.85 μm in the control group, 160.89 ± 7.55 μm in the BAPT 20 group, 187.22 ± 7.36 μm in the BAPT 40 group to 214.56 ± 9.26 μm in the BAPT 60 group (*P* < 0.05), respectively.

Furthermore, after 21 d in vitro culture, the mean diameter of follicles in the BAPT 40 group (252.00 ± 8.93 μm) and BAPT 60 group (290.78 ± 8.02 μm) were significantly higher than that of control group (204.89 ± 5.60 μm) and BAPT 20 group (222.78 ± 11.51 μm) (*P* < 0.05), which further indicated that BAPT treatment significantly enhanced the growth of isolated follicles in a dose-dependent manner.

### Hormonal assays

**Fig. 3:**
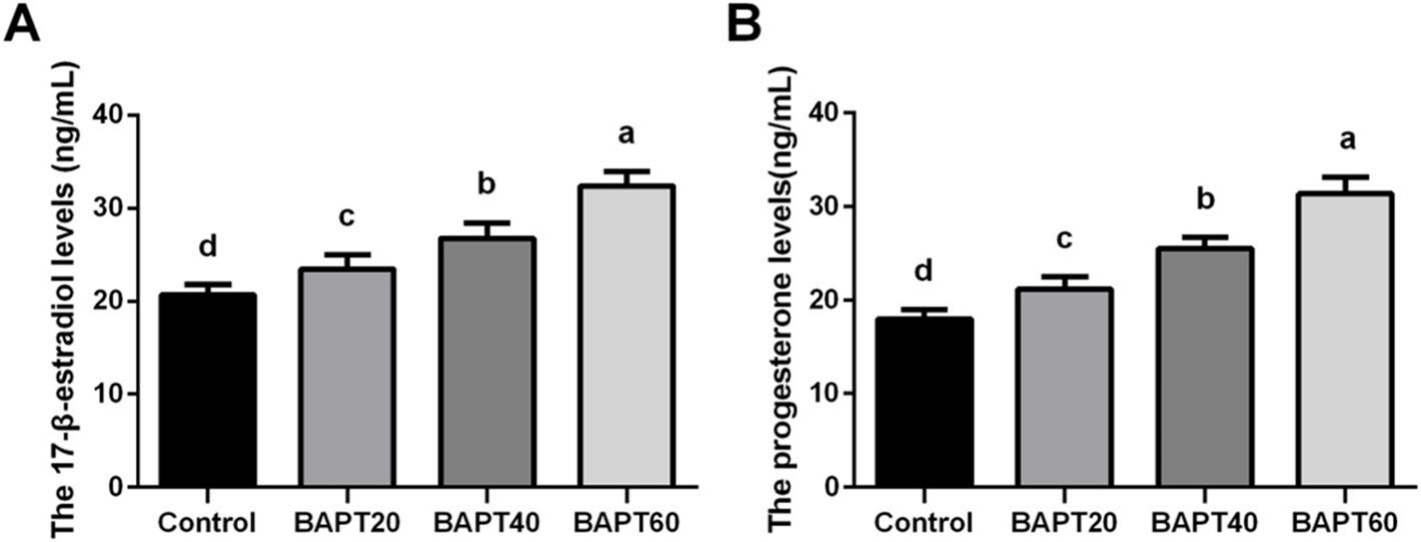
The hormone levels of follicles cultured in vitro for 14 d. A: E2 levels of follicles cultured in vitro after different periods; B: P4 levels of follicles cultured in vitro after different periods. Note: In each panel, labeling with the different letter in each column indicates significant differences between different groups (*P* < 0.05)

As shown in Fig. 3, after 14 d of in vitro culture, the level of E2 in the control group (20.70 ± 1.10 ng/mL) was significantly lower than those of BAPT treatment groups, respectively (*P* < 0.05). Indeed, the E2 levels were significantly increased from 23.45 ± 1.54 ng/mL in the BAPT 20 group, 26.73 ± 1.69 ng/mL in the BAPT 40 group to 32.41 ± 1.50 ng/mL in the BAPT 60 group (*P* < 0.05).

Furthermore, after 14 d in vitro of culture, the level of P4 significantly increased from17.98 ± 1.03 ng/mL in the control group, 21.23 ± 1.21 ng/mL in the BAPT 20 group, 25.51 ± 1.20 ng/mL in the BAPT 40 group to 31.40 ± 1.79 ng/mL in the BAPT 60 group (*P* < 0.05), respectively, which further showed that BAPT treatments significantly enhanced the functions of follicles in a dose-dependent manner.

### Gene expression patterns of in vitro cultured follicle

To assess the effect of BAPT treatment on the gene expression patterns of in vitro cultured follicle the expression levels of specific genes related to oogenesis (*Oct4*, *Bmp15*, *GDF9*, *FOXO3* and *Zp3*), granulogenesis (*FOXL2* and *Inhibin alpha*), ROS production (*SOD2*, *Catalase* and *GPx*) and apoptosis process (*BAX* and *Bcl-2*) in the developing follicles after 14 d of in vitro culture were analyzed by RT-PCR.

**Fig. 4:**
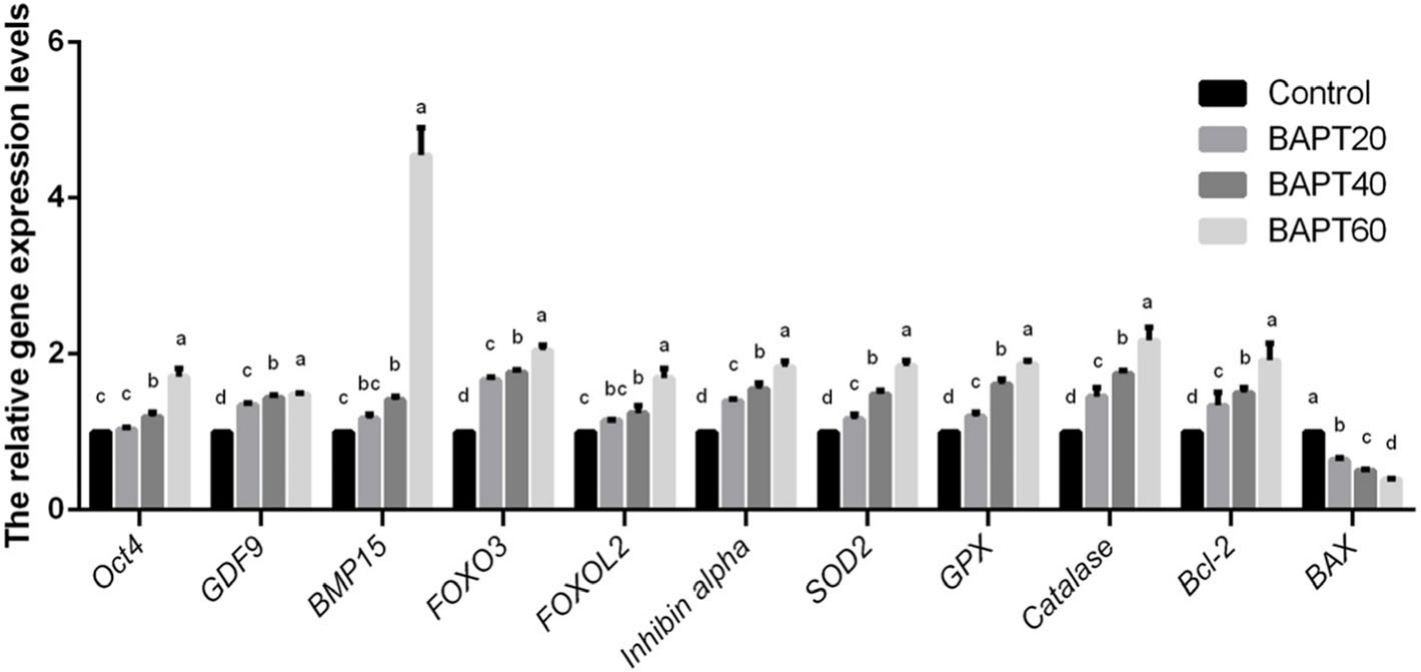
The relative gene expression levels in follicles cultured in vitro for 14 d. Note: Labeling with the different letter in each column indicates significant differences between different groups (*P* < 0.05)

As shown in Fig. 4, BAPT treatments significantly promoted the relative expression levels of specific genes related to oogenesis (*Oct4*, *Bmp15*, *GDF9*, *FOXO3* and *Zp3*) and granulogenesis (*FOXL2* and *Inhibin alpha*) in the developing follicles in a dose-dependent manner (*P* < 0.05).

In addition, the relative expression levels of ROS production-related genes (*SOD2*, *Catalase* and *GPx*) in BAPT treatment groups were significantly up-regulated with the increasing doses of BAPT (*P* < 0.05), indicating that BAPT treatment significantly inhibited ROS production of in vitro cultured follicles.

On the other hand, the relative gene expression levels of *BAX* in the BAPT 40 group and BAPT 60 group were significantly lower than that of control and BAPT 20 group (*P* < 0.05). However, after BAPT treatment, the relative gene expression levels of *Bcl-2* in all BAPT treated group were significantly higher than that in the control group (*P* < 0.05).

### The viability analyses of in vitro cultured follicles

**Fig. 5:**
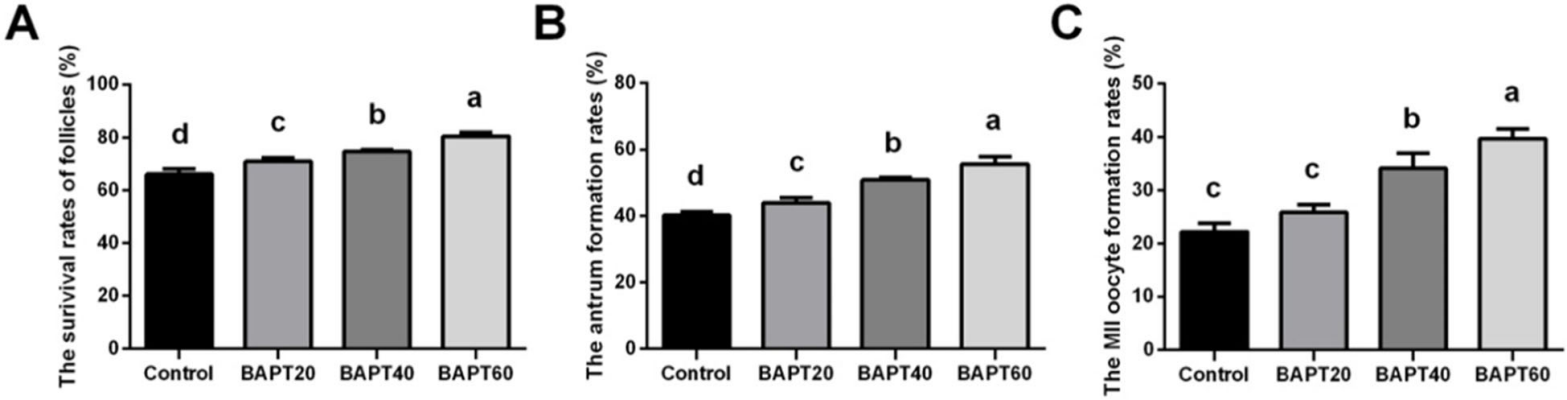
The developmental rates of follicles cultured in vitro for 21 d. A: The survival rates of follicles cultured in vitro for 21 d; B: The antrum formation rates of follicles cultured in vitro for 21 d; C: The MII oocytes developmental rates of follicles cultured in vitro for 21 d. Note: In each panel, labeling with the different letter in each column indicates significant differences between different groups (*P* < 0.05)

To investigate the effect of BAPT treatments on the viability of in vitro cultured follicles, the development of the follicles in different groups were analyzed by the measurement of viability rates, antrum formation rates and MII oocyte developmental rates calculated after 21 d of follicle culture. As shown in Fig. 5, the viabilities of follicles (Fig. 5A) and antrum formation rates (Fig. 5B) were significantly increased in the BAPT 40 group and BAPT 60 group in comparison with that of the control group and BAPT 20 group, respectively (*P* < 0.05).

After in vitro maturation, the developmental rate of MII oocytes (Fig. 5C) in the BAPT 60 group (39.70 ± 1.81%) was significantly higher than those of the control group (22.20 ± 1.66%), BAPT 20 group (25.84 ± 1.48%) and BAPT 40 group (34.08 ± 2.85%), respectively (*P* < 0.05).

### ROS production and mitochondrial distribution analyses of MII oocytes

**Fig. 6:**
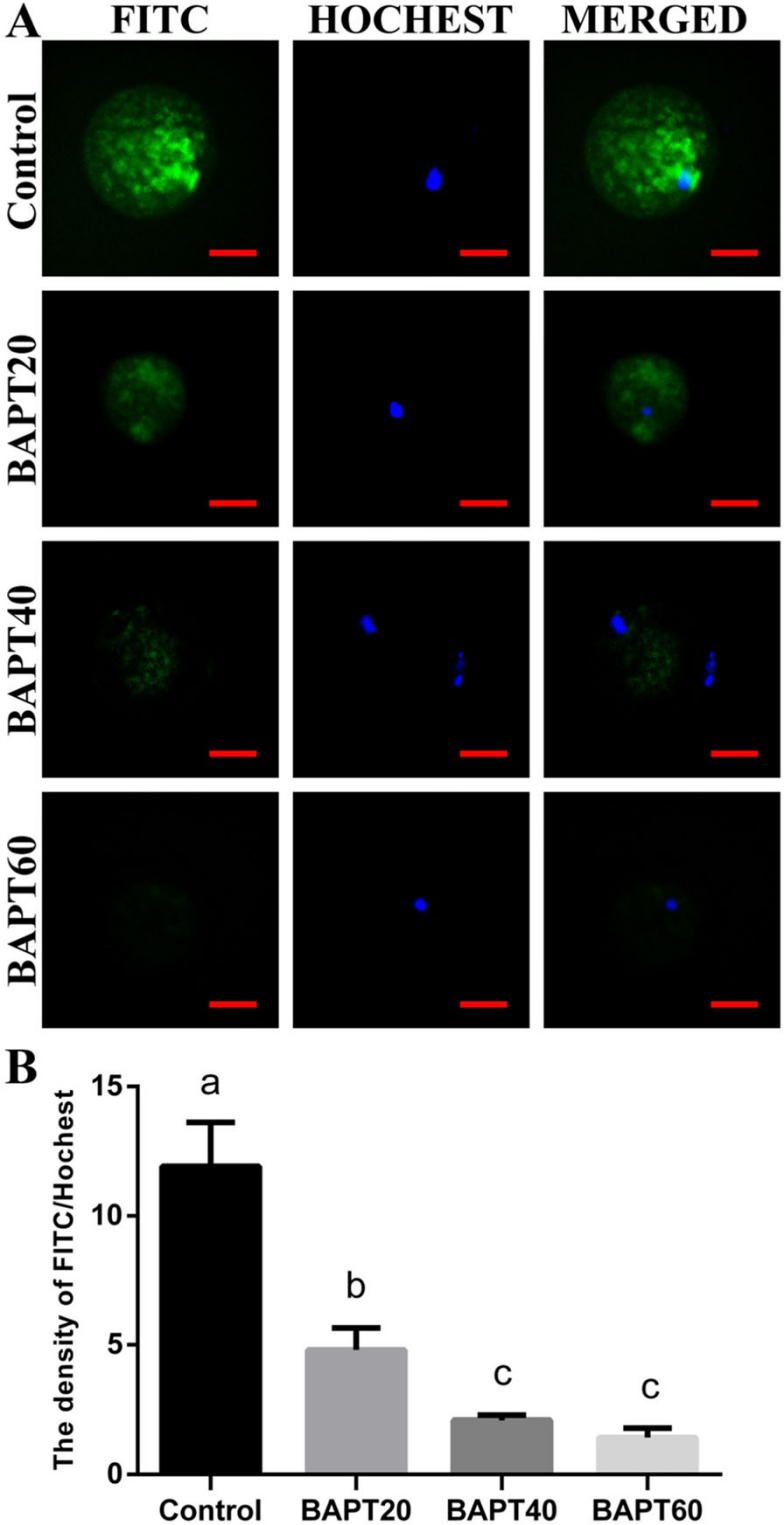
The ROS production levels in MII oocytes from different groups. A: The representative ROS staining of MII oocytes from different groups; Note: Scale bar = 30 μm. B: The relative density of FITC/ Hochest staining of MII oocytes from different groups. Note: Labeling with the different letter in each column indicates significant differences between different groups (*P* < 0.05)

**Fig. 7:**
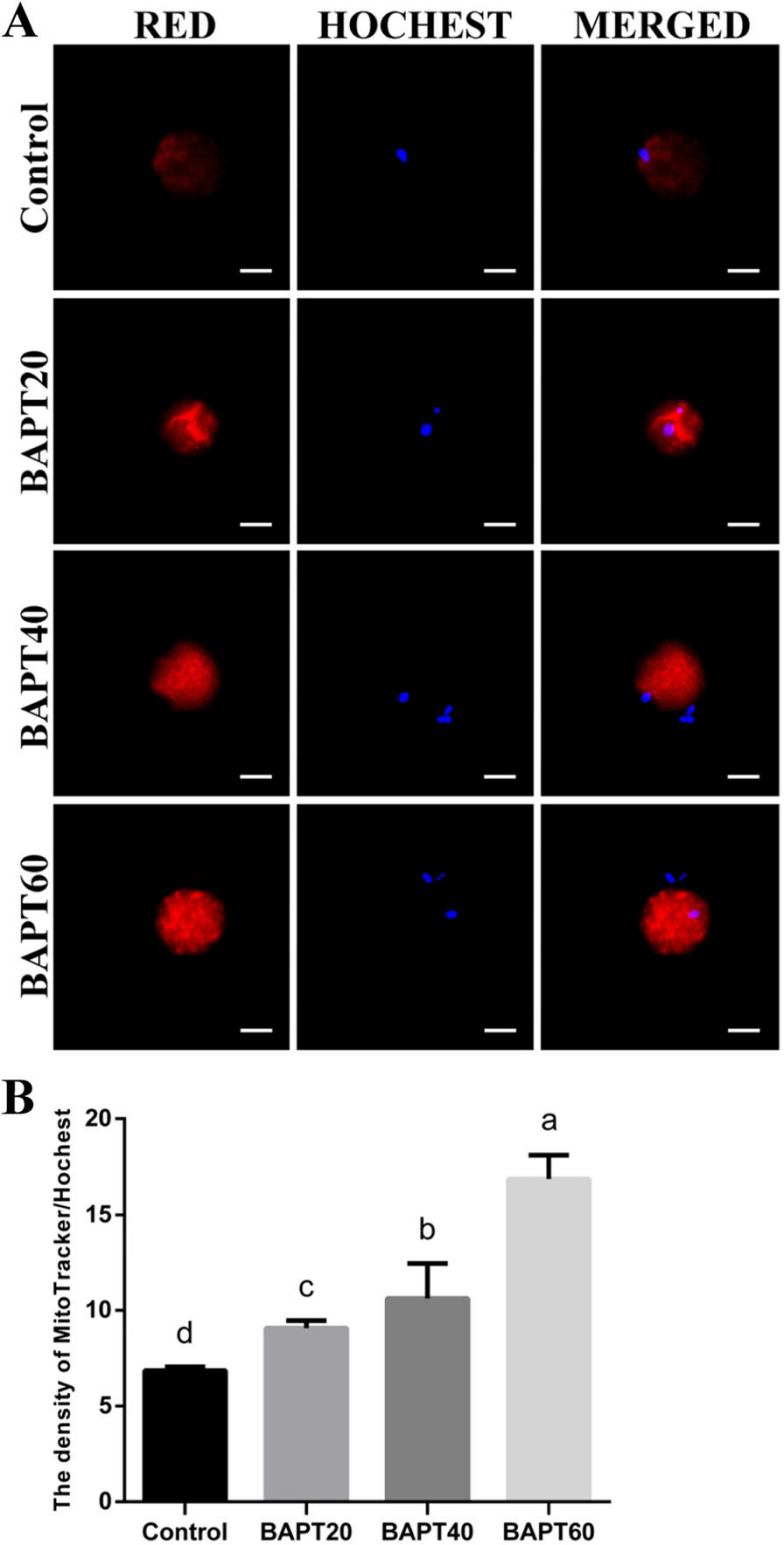
The mitochondrial distributions in MII oocytes from different groups. A: The representative mitochondrial staining results of MII oocytes from different groups; Note: Scale bar = 20 μm. B: The relative density of MitoTracker/Hochest staining of MII oocytes from different groups. Note: Labeling with the different letter in each column indicates significant differences between different groups (*P* < 0.05)

To investigate the effect of BAPT treatments on the viability of oocytes, ROS production levels and mitochondrial distributions in MII oocytes of different groups were analyzed with the representative images of DCFH-DA fluorescence staining and mitochondrial distribution in MII oocytes shown in Fig. 6 and Fig. 7, respectively.

As shown in Fig. 6, the fluorescence intensities of DCFH-DA in MII oocytes were remarkably decreased in all BAPT treated groups compared with the control group (*P* < 0.05), indicating that BAPT treatment significantly inhibit ROS production process in MII oocytes during the long-term in vitro culture process of follicles. Moreover, the reduction of fluorescence intensity of DCFH-DA in MII oocytes was negatively related with the increasing concentration of BAPT (*P* < 0.05).

Furthermore, the fluorescence intensities of MitoTracker Red CMXRos in MII oocytes were remarkably increased in all BAPT treated groups compared with the control group (*P* < 0.05) (Fig. 7), indicating that BAPT treatment significantly promoted the mitochondrial accumulation levels of MII oocytes during the long-term in vitro culture process of follicles.

### Fertilization rate and embryonic development analyses

**Fig. 8:**
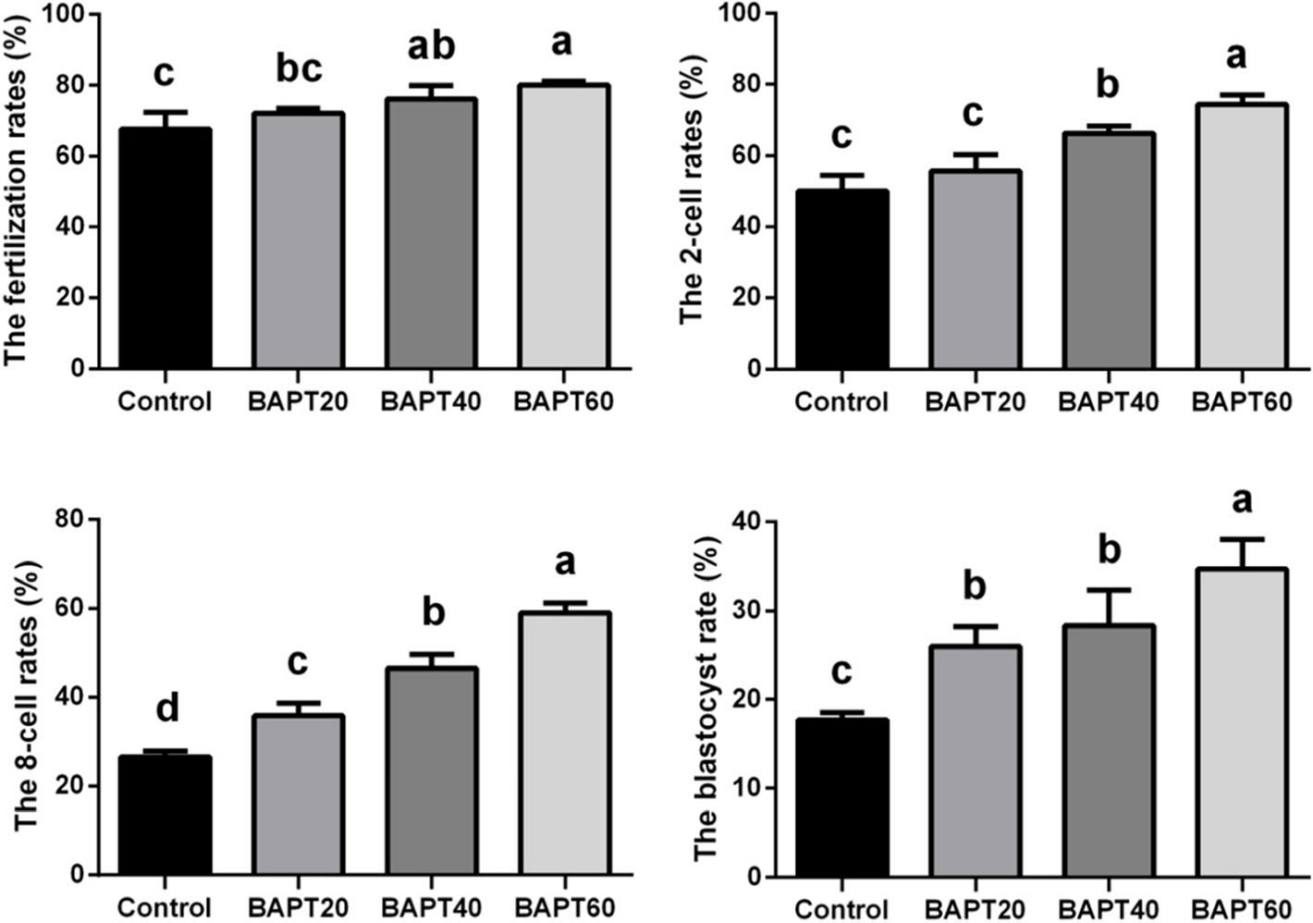
The fertilization rates and embryonic developmental rates in different groups. Note: Labeling with the different letter in each column indicates significant differences between different groups (*P* < 0.05)

The functional analysis of MII oocytes to develop into embryos after fertilization is the gold standard for the examination of in vitro follicular cultures. To analysis the developmental potential of MII oocytes, the fertilization rates of MII oocytes and embryonic developmental rates in different BAPT groups were further measured. As shown in Fig. 8, the percentage of fertilization rates was remarkably increased in all BAPT treated groups (*P* < 0.05) with the highest percentage of fertilization rate found in the BAPT 60 group. Moreover, the embryonic developmental rates from 2-cell embryo, 8-cell embryo to blastocyst were significantly higher in all BAPT treated groups compared with the control group (*P* < 0.05).

## Discussion

Here we provided the first evidence that BAPT promotes the growth of in vitro cultured preantral follicles and oocytes with remarkable up-regulation of oogenesis/granulogenesis process and significant inhibition of cellular ROS production and apoptosis.

Under normal conditions, the generation and elimination of ROS are effectively controlled by the cellular antioxidant defense system [33, 34], consisting of antioxidant enzymes (catalases in peroxisomes, glutathione peroxidases and superoxide dismutases in the cytosol), nonenzymatic factors (carnitine, coenzyme Q10, glutathione, pantothenic acid and vitamins) and micronutrients (copper, selenium and zinc) [35]. However, the unbalances between ROS production levels and the generation of free radicals under pathological conditions lead to oxidative damages to cellular proteins, membrane lipids and DNA, resulting in these health disorders including malignant cancer, coronary heart diseases and neuronal degeneration via dysregulted ATP generation, catabolic/anabolic processes and post-translational protein modifications [34, 36, 37]. Furthermore, the lipid oxidation mediated by excessive accumulation of free radicals interferes with these cellular amino acids, proteins, vitamins and cholesterol during the process of food preparation, transportation and storage, resulting into toxic compounds.

The increasing demand for effective antioxidants to delay the lipid oxidation in the pharmaceutical and health food industry promoted the development of antioxidants including butylated hydroxytoluene (BHT), butylated hydroxyanisole (BHA) and tertiary butylhydroquinone (TBHQ). However, these artificial antioxidants have been reported to be associated with liver damage and carcinogenesis. Therefore, the searching for a novel, natural and efficient antioxidant will promote the application of antioxidants in the functional and designer food industry.

During the promising investigation of natural antioxidants, BAPT have attracted increasing interest as prominent candidates. As a consequence, various products or ingredients with BAPT as fundamental constituents were commercially available and marketed globally, which were based on the specific characteristics of BAPT including anticancer [38], antimicrobial [39], antihypertensive [40], antithrombotic [41], antioxidative [42], immunomodulatory [43] and cholesterol-lowering properties [44]. These products are enriched with BAPT either by modification of the usual manufacturing processes or by simply adding the BAPT during product preparation. Such BAPT-based functional food market is a fast-growing segment of the food industry and is still expanding thanks to the increasing awareness and purchasing power of consumers.

In our study, BAPT was supplied as a natural antioxidant during in vitro culture of preantral follicles. We found that BAPT supplement significantly promoted the growth of in vitro cultured preantral follicles and oocyte qualities in a dose-dependent manner, as confirmed by the expression levels of reproduction-related genes in follicles and developmental rates of oocytes in vitro. Moreover, the growth promotion mechanisms were significantly associated with the reduction of ROS levels during follicle development.

Considering the growth of preantral follicles during the long-term culture process in vitro, the analyses of RT-PCR and hormone levels were conducted after 14 days of follicle culture [45, 46]. After BAPT treatment, the ROS production-related genes as *SOD2*, *Catalase* and *GPx*, which regulated the cellular antioxidant defense system, were significantly up-regulated with the increasing dose of BAPT, indicating that BAPT treatment significantly inhibited ROS production process of isolated follicles cultured in vitro.

During the past decades, numerous studies have confirmed that increased ROS levels were related with the disruptions of oocyte maturation and embryonic development through mitochondrial damages [47–49]. Therefore, to further confirm the effect of BAPT on the inhibition of ROS production in preantral follicles cultures, the mitochondrial distributions of MII oocytes were analyzed. We found that after BAPT treatment, higher APT synthesis was accompanied by the inhibition of ROS production, illustrating the function of BAPT in reducing ROS-dependent energetic impariments. As the ATP-generating organelles, mitochondria distributed around the nucleus play crucial roles in oocyte maturation, fertilization and early embryonic development. Increased mitochondrial distribution after BAPT treatment likely contributes to an enhanced meiotic progression of oocytes.

On the other hand, the increased expression levels of *Bcl-2* and decreased expression levels of *Bax* in follicles, which are considered as the biomarkers of inhibited cellular apoptosis process, were observed in BAPT-treated groups, demonstrating that BAPT treatment inhibit cellular apoptosis by regulating the expression of critical genes.

Therefore, the results of the present study have promising implications for biomedical research and warrant further in vivo studies. Future research should verify the role of BAPT in the prevention and treatment of other infertility disorders, such as PCOS, ovarian aging and clinical infertilities. In addition, the effect of BAPT on follicle’s gene expression and hormone expression levels after long-term culture process than 21 d needs further explorations.

## Conclusions

In conclusion, bioactive peptides (BAPT) from animal sources significantly improved follicular development throughout long-term in vitro culture of mouse preantral follicles*.* During the culture process, BAPT treatment reduced cellular damage caused by ROS production and promoted the mitochondrion levels in follicles with significantly increased maturation rates, fertilization and embryonic developmental rates in vitro*.*

## Methods

### Chemicals

Unless otherwise stated, all chemicals, media and supplements used in this study were purchased from Sigma Aldrich (Shanghai, China). The BAPT with the special characteristic of ubiquitin enzyme and molecular weight as 8Kd were derived from spleen of small-tailed-Han sheep and identified by Professor Su’s research group at Inner Mongolia Medical University (Hohhot, China) with the RRLC-MS method [22, 50–52].

Briefly, five small-tailed-Han sheep (2-year-old females; Inner Mongolia Dumei Animal Husbandry Biotechnology Co., Ltd) were housed at 22–24 °C with humidity at 45–50% and a natural light/dark cycle. Standard mineral blocks and water were offered to animals ad libitum. After animal sacrifice, the spleen was obtained and rapidly pulverized.

After pulverization, the spleen mixtures were homogenized with normal saline at a ratio of 0.125–0.5 g/mL, followed by frozen and thaw under − 80 °C for three times. After filtration, the supernatants were centrifuged under 4 °C at 14000×g for 20 min. After centrifugation, the supernatants were collected and BAPT was isolated with the RRLC-MS method.

Animals, follicle isolation and experimental design.

A total of 50 6-week-old females mice of the Institute of Cancer Research (ICR) strain (Beijing Vital River Laboratory Animal Technology Co., Ltd., 201) were used for this study. The ICR mice were housed in a controlled environment at 21 °C, 53 to 63% relative humidity with 12-h light/dark cycles. Standard feed and water were provided ad libitum.

The ICR mice were sacrificed by cervical dislocation; subsequently, bilateral ovaries were dissected and placed immediately into fresh medium as *αlpah*-minimal essential medium (α-MEM) (12,571,063, Gibco, Shanghai, China) supplemented with 10% fetal bovine serum (FBS) (10099141C, Gibco, Shanghai, China). The follicles were isolated mechanically under a stereomicroscope (745 T, Nikon, Tokyo, Japan) with a 26-gauge needle, then intact preantral follicles displaying two or three layers of granular cells (GC) with normal and centrally located oocytes were collected [53]. These collected preantral follicles were rinsed twice and transferred into a culture dish with fresh basic culture medium.

The preantral follicles (*n* = 1198) were randomly divided into the following four groups based on the dose of supplemented BAPT to the basic culture medium. The control group (*n* = 289) was not treated with any BAPT, while the BAPT 20 (*n* = 310), BAPT 40 (*n* = 297) and BAPT 60 groups (*n* = 302) were supplemented with 20, 40 and 60 μg/mL BAPT, respectively. The optimization of BAPT concentration applied in this study was performed in our former study [50].

### In vitro culture of preantral follicles

The isolated preantral follicles were cultured on an insert (PICM0RG50, Millipore, USA) in 6-well culture dishes (Corining, China) in 1200 μL culture medium as α-MEM medium supplemented with 2% FBS, 1 × Glutamax (35,050,061, Gibco, Shanghai, China), 0.055 mM 2-mercaptoethanol (M6250, Sigma, Shanghai, China), 100 IU/mL follicle stimulating hormone (FSH) (Sansheng**,** Ningbo, China) and 100 IU/mL penicillin/streptomycin (P/S, 15140122, Gibco, Shanghai, China) for 21 d in a CO_2_ incubator (37 °C and 5% CO_2_). Half of the culture medium was replaced by fresh culture medium every other day.

After in vitro follicle culture for different periods (7 d, 14 d and 21 d), the morphologies of the cultured follicles were examined and recorded with an inverted fluorescence microscope (Ti2, Nikon, Tokyo, Japan). Images of the cultured follicles were randomly taken for each group at a 10× magnification. The mean diameters of the developing follicle were analyzed through Image J software (*n* = 10 for each group).

### Hormonal assays

After in vitro follicle culture for 14 d, half of each follicle culture medium was collected to investigate the sex hormone levels of 17β-estradiol (E2) and progesterone (P4) with a commercial estradiol ELISA kit (PE223, Beyotime, Shanghai, China) and a commercial progesterone ELISA kit (PP773, Beyotime, Shanghai, China), respectively.

### Reverse transcription PCR and real-time PCR analyses

After in vitro follicle culture for 14 d, total RNA from cultured follicles (*n* = 30) of different groups was extracted with Trizol reagent (79,306, Gibco, Shanghai, China), respectively. The synthesis of cDNA was conducted according to the manufacturer’s instructions (Prime Script™ RT reagent kit with gDNA Eraser, RR047A, Takara, Dalian, China). For PCR amplification, specific primers were designed on the NCBI website and commercially synthesized by Invitrogen (Shanghai, China). Real-time PCR was performed within a Thermo Scientific PikoReal system with a commercial kit (RR820A, Premix Taq™, Takara, Dalian, China).

PCR reactions started with an initial melting step for 5 min at 95 °C, followed by 35 cycles of melting at 95 °C for 30 s, annealing at 58 °C for 30 s and elongation at 72 °C for 30 s. The ubiquitously expressed *β-actin* gene was used as an internal control. The qualities of PCR reactions were confirmed by the melting curve analyses with all experiments performed in triplicate. The relative mRNA expression was calculated using the 2^-ΔΔCt^ method [54].

The primers for Reverse Transcription PCR and Real-time PCR analyses were as follows:

*Oct4.*

Forward primer-*GATGCTGTGAGCCAAGGCAAG*;

Reverse primer-*GGCTCCTGATCAACAGCATCAC*;

*Bmp15.*

Forward primer-*TCCTTGCTGACGACCCTACAT*;

Reverse primer-*TACCTCAGGGGATAGCCTTGG*;

*GDF9.*

Forward primer-*TCTTAGTAGCCTTAGCTCTCAGG*;

Reverse primer-*TGTCAGTCCCATCTACAGGCA*;

*FOXO3.*

Forward primer-*CTGGGGGAACCTGTCCTATG*;

Reverse primer-*TCATTCTGAACGCGCATGAAG*;

*FOXL2.*

Forward primer-*ACAACACCGGAGAAACCAGAC*;

Reverse primer-*CGTAGAACGGGAACTTGGCTA*;

*Inhibin alpha.*

Forward primer-*GCACAGGACCTCTGAACCAG*;

Reverse primer-*GGGATGGCCGGAATACATAAG*;

*SOD2.*

Forward primer-*ATGGTGGGGGACATATT*;

Reverse primer-*GAACCTTGGACTCCCACAGA*;

*Catalase.*

Forward primer-*CCTCGTTCAGGATGTGGTTT*;

Reverse primer-*TCTGGTGATATCGTGGGTGA*;

*GPx*

Forward primer-*GTCCACCGYGTATGCCTTCT*;

Reverse primer-*TCTGCAGATCGTTCATCTCG*;

*BAX*

Forward primer-*TGAAGACAGGGGCCTTTTTG*;

Reverse primer-*AATTCGCCGGAGACACTCG*;

*Bcl-2.*

Forward primer-*ATGCCTTTGTGGAACTATATGGC*;

Reverse primer-*GGTATGCACCCAGAGTGATGC*;

*β-actin.*

Forward primer-*GGCTGTATTCCCCTCCATCGT*;

Reverse primer-*TGGTGCCAGATCTTCTCCATGTC*.

### In vitro maturation

After in vitro follicle culture for 21 d, the developing follicles of each group were mechanically dissected by with a pulled bore glass capillary with the released cumulus cell-oocyte complexes (COC) collected for in vitro maturation (IVM).

The collected COCs were then cultured in a 4-well culture dish (Nunc, China) in 500 μL α-MEM medium supplemented with 5% FBS, 0.1 IU/mL FSH, 1.2 IU/mL human chorionic gonadotropin (HCG, Sansheng, Ningbo, China), 4 ng/mL epidermal growth factor (EGF, 315–09, Proteintech, Shanghai, China), 1.5 mM ascorbic acid (A4403, Sigma, Shanghai, China) and 100 IU/mL P/S covered in mineral oil (M8410, Sigma, Shanghai, China) in a CO_2_ incubator (37 °C and 5% CO_2_).

After 17 h of culture, oocytes from the expanded COCs were further examined microscopically for the maturation with first polar bodies. The maturation rates of each group were further analyzed with the maturation rates calculated as the percentage of maturing oocytes for each developing follicles [54].

### Analyses of ROS levels in MII oocytes

MII oocytes from different groups were collected and incubated in CZB medium containing 10 μM dichlorofluorescein diacetate (DCFH-DA, S0033, Beyotime, Shanghai, China) for 30 min at 37 °C according to the manufacturer’s protocols. After incubation, MII oocytes were re-stained with 5 μg/mL Hoechst 33432 solution (C0030, Solarbio, Beijing, China) for 10 min at 37 °C. The fluorescence staining density of oocyte were analyzed by confocal microscopy (A1R, Nikon, Tokyo, Japan).

Mitochndrial distribution analyses of MII oocytes.

MII oocytes from different groups were collected, rinsed three times with Dulbecco’s phosphate buffered solutions (DPBS, 14190136, Gibco, Shanghai, China) and incubated in 4% paraformaldehyde solution (P1110, Solarbio, Beijing, China) at room temperature for 30 min, subsequently stained with 200 nM Mito-Tracker Red CMXRos (C1049, Beyotime, Shanghai, China) for 30 min at 37 °C according to the manufacturer’s protocols. After incubation, MII oocytes were further re-stained with 5 μg/mL Hoechst 33432 solution at room temperature for 10 min and mounted on microscope slides for subsequent confocal microscopy.

### IVF and embryo culture

To perform in vitro fertilization (IVF), the cauda epididymis of male ICR mice (ten weeks old) was dissected and placed immediately into the human tubal fluid (HTF) medium supplemented with 4 mg/mL BSA for 1 h in a CO_2_ incubator (37 °C and 5% CO_2_) for sperm capacitation. After sperm capacitation, the collected MII oocytes from different groups were transferred to HTF medium containing 1–2.5 × 10^6^ capacitated spermatozoa and incubated for 6 h in a CO_2_ incubator (37 °C and 5% CO_2_). After insemination, the embryos were transferred to the Embryo^Max^ KSOM medium (MR-020P-D, Sigma, Shanghai, China), which was supplemented with 1 mg/mL of BSA and covered by mineral oil in a 35 mm sterile plate (Corning). The embryos were cultured in vitro for 3.5 d. During this period, the embryonic developmental stages (2-cell embryo, 8-cell embryo and blastocyst stage) of different groups were microscopically observed and recorded with the embryonic development rates calculated as percentage of embryo/fertilized oocytes.

#### Statistical analysis

The analyses of results were carried out with the Statistical Package for the Social Sciences (SPSS, IBM, version19.0). All results are presented as mean ± SD and assessed with one-way ANOVA LSD tests. *P* < 0.05 was considered as significant.

## Data Availability

We declared that materials described in the manuscript, including all relevant raw data, will be freely available to any scientist wishing to use them for non-commercial purposes, without breaching participant confidentiality.

## Abbreviations

BAPT: Bioactive peptides
BHA: Butylated hydroxyanisole
BHT: Butylated hydroxytoluene
COC: Cumulus cell-oocyte complex
DSB: DNA double strand break
FBS: Fetal bovine serum
FSH: Follicle stimulating hormone
GC: Granular cells
GFC: Gel filtration chromatography
HTF: Human tubal fluid
IEX: Ion-exchange chromatography
IVF: In vitro fertilization
IVM: In vitro maturation
OS: Oxidative stress
PCOS: Polycystic ovary syndrome
ROS: Reactive oxygen species
RRLC-MS: Rapid resolution liquid chromatography-tandem mass spectrometry
SPSS: Statistical Package for the Social Sciences
TBHQ: Tertiary butylhydroquinone
UHPLC-MS/MS: Ultra-high-performance liquid chromatography-tandem mass spectrometry
